# Translation and psychometric properties of the Mandarin Chinese version of the COVID-19 Impact Scale in college students

**DOI:** 10.3389/fpsyt.2023.1267943

**Published:** 2023-10-10

**Authors:** Qing Zhang, Yahui Liu, Jinxin Yang, Chengwei Liu, Haoyu Yin

**Affiliations:** ^1^College of Politics and Public Administration, Shandong Youth University of Political Science, Jinan, China; ^2^School of Mechanical Engineering, University of Jinan, Jinan, China; ^3^School of Physical Education, Shandong Youth University of Political Science, Jinan, China

**Keywords:** COVID-19 pandemic, scale development, factor analysis, reliability, validity

## Abstract

The COVID-19 pandemic has caused significant long psychological impacts that require a novel measurement tool to capture the changes in such impacts. To this end, the COVID-19 Impact Scale (CIS) was developed as an instrument to evaluate psychological responses associated with the pandemic, and has shown evidence of a one-factor structure. The CIS was initially created using an Korea University students sample, and has since been translated and validated in Turkish. A total of 504 College students, aged 17–25, took part in the study from two universities in Jinan, located in Shandong Province, Eastern China, via an online survey platform. They were administered the Chinese versions of the following self-report instruments: Mandarin Chinese CIS, Fear of COVID-19 Scale, Depression Anxiety Stress Scale-21 and Satisfaction With Life Scale. Moreover, a sample of 86 participants who provided their contact information and agreed to participate in the second-round survey were asked to reassess using the Mandarin Chinese CIS after a period of 3 weeks following the initial testing. Results showed that Mandarin Chinese CIS had good internal consistency and test–retest reliability. Additionally, the Mandarin Chinese CIS presented good criterion validity and estimates of convergent validity and incremental validity. In confirmatory factor analysis, the one-factor model showed an acceptable fit after incorporating correlations between error terms. Our findings suggest that the Mandarin Chinese CIS is a reliable and valid self-report tool that demonstrates robust psychometric properties and acceptable construct validity when used with a Chinese university students.

## Introduction

Epidemic viruses have had a profound impact on human health throughout history. From the SARS to Ebola to COVID-19, these highly infectious agents have not only caused widespread illness and death but also profound psychological impacts on individuals and communities. COVID-19 was first detected in December 2019 in Wuhan, China. Shortly afterward, it quickly spread across the globe, becoming a pandemic by March 2020. On September 21th, 2023, there were 770,778,396 confirmed cases of COVID-19 and 6,958,499 deaths in the world (1), while as of September 21th, 2023, China had recorded 99,309,232 confirmed cases and 121,679 deaths (2). Although the World Health Organization (WHO) has declared that the COVID-19 pandemic is no longer a “public health emergency of international concern” (3), recent data from the Beijing Center for Disease Control and Prevention (CDC) shows that COVID-19 has surpassed influenza as the most frequently reported infectious disease in China since the 37th week of 2023 (September 11–17) (4). Therefore, the COVID-19 remains a highly infectious disease, at least in China.

In fact, the COVID-19 pandemic has highlighted the significant impact of epidemics on mental health. Studies show that individuals are experiencing anxiety, depression, and stress (5, 6). Certain populations, such as healthcare workers (7, 8), older adults (9, 10), children and adolescents (11, 12), low-income communities (13), and those with preexisting mental health disorders (14, 15) are particularly vulnerable to the psychological consequences of the pandemic. However, it is undeniable that university students are indeed one of the groups heavily affected by the adverse consequences of the pandemic. Studies have shown that COVID-19 has posed challenges for university students in various areas, such as social participation, sense of achievement, pursuit of goals, and academic advancement, in addition to the uncertainty it has brought (16, 17). A meta-analysis conducted on literature from 2020 to 2021 revealed that 26.0% (95%CI: 23.3–28.9%) of university students experienced depressive symptoms (18). Another meta-analysis study, conducted on literature published prior to March 28, 2021, also found that the prevalence of depression and anxiety symptoms among college students was found to be 33.6% (with a 95% confidence interval [CI] of 29.3–37.8%) and 39.0% (with a 95% CI of 34.6–43.4%), respectively (19).

In order to prevent or/and reduce the occurrence of psychological problems, it is important to understand what negative psychological disorders that individuals are experiencing during the pandemic (20–25). In fact, several questionnaires have been developed and validated across different countries to measure psychological effects of COVID-19. These include the Fear of COVID-19 Scale (26), the COVID-19 anxiety syndrome scale (27), the COVID-19 Stress Scales (28) and the COVID-19 Perceived Risk Scale (29) and so on. The measures focus on assessing various aspects such as fear, depression, anxiety, stress in relation to COVID-19. Some scales, such as the Fear of COVID-19 Scale, are widely used in China (30, 31). Some researchers, however, have proposed that the majority of scales developed in the early phases of the pandemic primarily measure acute stress responses that were commonly observed at that time (32, 33). Therefore, it is imperative to regularly verify the reliability and validity of assessment tools specifically designed for the university student population within the general community. These tools should accurately reflect the current characteristics of the pandemic and be easily applicable to the unique needs.

The COVID-19 Impact Scale (CIS), developed in 2022, is a self-report measure that specifically assesses symptoms of multiple disorders (32). It comprises of 10 items and has been found to exhibit a single-factor structure. The CIS has an excellent reliability in terms of internal consistency and the convergent and divergent validity. The CIS was initially created using an Korea University students sample (*N* = 2,152), and has since been translated and validated in Turkish (33) and Spanish (34). The CIS has several advantages. Firstly, it is a valid and reliable scale and demonstrates better explanatory power compared to existing scales. For instance, research has found that the CIS has incremental validity even after controlling for the FCV-19S scale (32). Secondly, the CIS effectively encompasses a broader spectrum of emotional reactions and functional difficulties despite its brevity, enhancing its comprehensiveness in measuring the impact of the COVID-19 pandemic. Scales developed during the early stages of the pandemic primarily emphasized pathological responses, such as fear (26) and anxiety (27). In summary, the CIS is a valuable tool for research and practical applications due to its superior explanatory power of COVID-19 effects and comprehensive assessment.

Accordingly, the purpose of this study was to develop a Mandarin Chinese version of the CIS and assess its factor structure, internal consistency, test–retest reliability, along with its validity. To that end, the three aims of Study were as follows. Firstly, the CIS was translated into Mandarin Chinese. Secondly, its factor structure was tested. It is expected that the Mandarin Chinese version of the CIS possess a unidimensional structure similar to those established in the previous study (32, 33). Thirdly, its internal consistency reliability, test–retest reliability, and convergent validity, criterion validity and incremental validity were estimated. It is expected that its provide a reliable estimate and convergent validity. Additionally, a positive correlation between the CIS and FCV-19S, along with negative affect, is predicted, while a negative correlation with subjective well-being is expected for criterion validity. Moreover, the CIS is hypothesized to uniquely predict psychological distress after controlling for FCV-19S for incremental validity.

## Methods

### Participants

The questionnaire and methodology for this study were approved by the Institutional Review Board at Shandong Youth University of Political Science. A non-probability sampling strategy was adopted. The research team selected two universities in Jinan, Shandong Province, and reached out to counselors at these universities for assistance. Afterwards, a hyperlink to an online survey was sent to the counselors who accepted the research team’s invitation, and they further distributed the hyperlink to their students. A total of 513 university students took part in the study.

### Procedure

All participants via an online survey platform^1^ from May 11–24, 2023. Prior to participating in the study, individuals were presented with an electronic informed consent form on the webpage. At the bottom of the page, there were two buttons available: “I agree” and “I disagree.” By clicking “I agree,” individuals indicated their acceptance of the informed consent and would be considered regular participants. Conversely, selecting “I disagree” indicated their refusal to participate, and they would not be chosen as participants. Data from 9 participants were removed due to invalid responses. Of the remaining 504 (378 women) participants, age ranged from 17 to 25 years (*M* = 19.35 years, SD = 0.68). For the test–retest reliability analysis, 86 participants (72 women) who provided their anonymous contact information and agreed to participate in the second-round survey. They were asked to reassess using the Mandarin Chinese CIS after a period of 3 weeks following the initial testing. To ensure consistency between the two evaluations for the same participant, the participant provided a fictitious ID number during the first assessment, which was then maintained and used in the second assessment.

### The COVID-19 Impact Scale

The COVID-19 Impact Scale (32) is a self-reported measurement consisting of 10 items. It includes the assessment of the general effect of COVID-19 related problems on one’s current life (e.g., “To what extent is your current life affected by COVID-19 related problems?”), concerning and stressful reactions (e.g., “How frequently do you experience stress related to COVID-19 problems currently?”), negative emotional reactions (e.g., “How often do you experience anger regarding COVID-19 related problems currently?”), and the interpersonal and functional effects of COVID-19 related problems (e.g., “To what extent do COVID-19 related problems interfere with your interpersonal relationships?”). Participants were required to rate each item on a 5-point Likert scale, ranging from 0 (none) to 4 (very severe/very often). Excellent internal consistency reliability (*α* = 0.90) was reported for the CIS from original study.

### The development of the Mandarin Chinese CIS

First, The English version of the CIS was translated into Mandarin Chinese by two independent and bilingual Master’s students in English. The translated version was then back-translated into English by a English major teacher. Finally, both versions were reviewed by two associate professors at the Institute of Psychology who are fluent in Chinese and English. The translated version was ended after adding very minor changes.

### The Fear of COVID-19 Scale

The Chinese version of the Fear of COVID-19 Scale (FCV-19S) (30, 31) was composed of 7 items measuring individuals’ fear toward COVID-19 pandemic. All items are rated on a 5-point Likert scale, ranging from 1 (strongly disagree) to 5 (strongly agree). The Cronbach’s *α* for the was 0.856 in the present study.

### Depression Anxiety Stress Scale-21

The Depression Anxiety Stress Scale-21 (DASS-21) is a 21-item self-report scale assessing depression, anxiety and stress of negative affect states over the last week. Twenty one items employ a 4-point scale (1 = did not apply to me at all, 4 = applied to me very much or most of the time) to rate the frequency and severity of behavior. The Chinese version of the DASS-21 has been validated (35). Cronbach’s *α* of 0.931 indicated an excellent reliability in the present study.

### Satisfaction With Life Scale

The five-item Satisfaction With Life Scale (SWLS) (36) was used to assess the quality of participants’ lives. Each item was rated on a 7-pointscale ranging from ranging from strongly disagree to strongly agree. The SWLS has shown good psychometric properties among Chinese (37). In this study, Cronbach’s *α* was 0.830.

## Data analysis

Cronbach’s *α* coefficient was used to assess the internal consistency of the Mandarin Chinese CIS, while test–retest reliability was evaluated using Intraclass correlation coefficients (ICCs). Shoukri, Asyali, and Donner proposed that sample sizes of 55 or more are sufficient to achieve a reliability level above 0.8 at a significance level of 0.05 with 80% power (38). Our sample size (*n* = 86) was therefore acceptable for conducting test–retest reliability based on these criteria.

Lavaan package with R studio was used to conducted CFA (39). Since each of scores was normally distributed (skewness values <3, kurtosis values <8) (40), parameters were performed by using the maximum likelihood estimation method. Previous study suggested that the sample should have a minimum size of 100 or/and the ratio of participants per parameter estimated (e.g., items or factors) should be at least 5:1, but ideally higher (41). Therefore, our sample sizes (N = 504) met both criteria for conducting CFA. Model fit was assessed using chi-square, comparative fit index (CFI), Tucker-Lewis fit index (TLI), standardized root mean square residual (SRMR) and root mean square error of approximation. As recommended by Hu and Bentler (42), models that meet the following criteria demonstrate a good fit to the data:CFI of 0.95 or higher, SRMR of 0.08 or lower, and RMSEA of 0.06 or lower. Meanwhile, models that show CFI between 0.90 and 0.94, SRMR between 0.09 and 0.10, and RMSEA between 0.07 and 0.10 are considered to have an acceptable fit.

Criterion validity was tested by examining Mandarin Chinese CIS relationship with the FCV-19S and DASS-21 and the SWLS. Pearson correlation coefficients were calculated. Moreover, regression analyses were conducted to examine whether the Mandarin Chinese CIS contributed independently to the prediction of negative affect states measured by DASS-21 after adjustment for gender, age and FCV-19S. Components that were able to predict the change of psychological symptoms were regarded as having incremental validity. Finally, convergent validity was assessed through construct reliability (CR) and average variance extracted (AVE).

## Results

### Descriptive statistics and reliability

Results on the descriptive statistics and internal consistency of the Mandarin Chinese CIS in this study are presented in Table 1. The data of all the Mandarin Chinese CIS item scores were confirmed to meet the assumption of normality. The results showed that Cronbach’s α for Mandarin Chinese CIS was 0.893. As shown in Table 1, the range of the Cronbach’s alpha if item deleted and item-scale correlation ware 0.873–0.892 and 0.513–0.766, respectively, indicating an acceptable reliability.

**Table 1:** Descriptive statistics and factor loading for the items of CIS (*n* = 504).

Item	Mean	SD	Skew	Kurt	CD	*r*_tot_	SFL
Item1	1.40	0.78	0.36	0.40	0.890	0.521	0.511***
Item2	1.27	0.83	0.33	0.16	0.885	0.590	0.505***
Item3	1.27	0.81	0.25	−0.27	0.885	0.592	0.656***
Item4	0.89	0.76	0.54	0.06	0.878	0.699	0.792***
Item5	0.82	0.84	0.84	0.31	0.876	0.726	0.836***
Item6	0.51	0.74	1.35	1.23	0.879	0.690	0.907***
Item7	0.73	0.85	1.08	0.96	0.873	0.766	0.802***
Item8	0.65	0.84	1.25	1.30	0.878	0.698	0.568***
Item9	0.84	0.86	0.79	0.05	0.886	0.575	0.434***
Item10	1.27	0.93	0.53	0.12	0.892	0.513	0.511***

SD, standard deviation; Skew, skewness; Kurt, kurtosis; CD, Cronbach’s alpha if item deleted; *r*_tot_, corrected item-total correlation, SFL, standardized factor loading, ***p < 0.001.

### Test–retest reliability

To assess the test–retest reliability of the Mandarin Chinese CIS, a second sample consisting of 86 university students was reevaluated after a three-week interval from the initial data collection. The Mandarin Chinese CIS demonstrated a test–retest reliability coefficient of 0.640. These findings provide support for the acceptable test–retest reliability of the Mandarin Chinese CIS.

### Confirmatory factor analysis

CFA was performed on Mandarin Chinese CIS to examine how well the one-factor structure fit the present data. Results indicated an inadequate goodness of fit, *χ*^2^(31) = 478.85, *p* < 0.001, CFI = 0.833, TLI = 0.786, SRMR = 0.084, and RMSEA = 0.159. In order to improve the model, modification indices were examined to determine whether additional paths could be added to the model. As a result of checking the modification indices, it was found that adding correlations between the error terms of items 1–2, 3–4, 4–5, and 9–10 would improve the model fit. In fact, these pairs of items have similar content, so there is theoretical support for these statistical findings. After adding these correlation terms, the results showed that the single factor solution provided an acceptable fit, *χ*^2^(31) = 159.99, *p* < 0.001, CFI = 0.952, TLI = 0.930, SRMR = 0.064, and RMSEA = 0.091 [90% CI: (0.077, 0.1050)]. Standardized factor loadings were all significant while ranged from 0.434 to 0.907 (see Table 1).

With regard to convergent validity, the CR value was found to be 0.856, and AVE was 0.461.

### Criterion validity

The Mandarin Chinese CIS was found to have a significant positive correlation with FCV-19S (*r* = 0.480, *p* < 0.001) and negative affect (*r* = 0.310, *p* < 0.001), and it was negatively correlated with subjective well-being (*r* = −0.160, *p* < 0.001). These results provided evidence of the criterion validity of the Mandarin Chinese CIS.

### Incremental validity

The Mandarin Chinese CIS demonstrated incremental validity even after controlling for variances in gender, age, and FCV-19S. In the first step, gender (a dummy code 1 for female), age (replace missing values with the mean value) and the FCV-19S were entered into the regression model. In the second step, Mandarin Chinese CIS was entered. Results showed that the Mandarin Chinese CIS produced a significant increase in variance that accounted for negative affect states in Step 2 (Δ*R*^2^ = 0.06, *p* < 0.001), and the regression coefficient of the CIS was significant (*β* = 0.27, *p* < 0.001). These results suggested that the CIS demonstrated good incremental validity.

## Discussion

As far as we know, the CIS has only been tested for psychological properties in Korea and Turkey (32, 33). Therefore, cross-cultural and linguistic validation studies are needed. The CIS was translated into Mandarin Chinese and its psychometric properties were examined in the study. As expected, the psychometric integrity of the Mandarin Chinese CIS was supported by our findings. This conclusion was based on three main criteria.

The first criterion was the reliability of the Mandarin Chinese CIS. As for the test–retest reliability, a ICC value of 0.60–0.80 indicates good test–retest reliability, and a value above 0.80 is considered excellent test–retest reliability (43). The study found good test–retest reliability (0.640), providing support for temporal reliability of the Mandarin Chinese CIS. Regarding internal consistency reliability, Cronbach’s α lower than 0.60 were considered poor, values between 0.70 and 0.80 acceptable, and values over 0.80 indicated good reliability (44). Mandarin Chinese CIS demonstrated excellent internal consistency (0.89), which is in consistent with the findings of previous research (32, 33).

The second criterion was the validity of the Mandarin Chinese CIS. For measures assessing criterion validity, Pearson correlation coefficients between 0.50 and 1.00, between 0.030 and 0.50, and < 0.30 were considered large, moderate and small, respectively (45). As such, the criterion validity of the Mandarin Chinese CIS was supported by moderate positive correlations with negative affect, as well as small negative correlations with subjective well-being. These correlations were in the expected direction and had magnitudes that were very similar to those found in the original study where the CIS was developed (32). Of note, previous research has not found correlation between CIS and subjective well-being. The discrepancy between our findings and those of Min et al. could potentially be attributed to differences in sample characteristics, which may influence the outcomes of the same variable (33). When assessing incremental validity, regression analyses indicated that the Mandarin Chinese CIS had incremental validity in predicting negative affect beyond other factors such as gender, age, and FCV-19S. This suggests that the CIS provided unique information and explained a significant portion of variance that other factors did not account for. This finding is consistent with previous research (32, 33). To evaluate convergent validity, CR with a recommended value of 0.70 or higher and AVE with a recommended value of 0.50 or higher were used (46). the AVE value in the current study was below cut-off points (0.50), but the CR value was ≥0.80. some researcher propose that if the AVE value is less than 0.50, but the CR value is equal to or greater than 0.80, the convergent validity can still be considered acceptable (47).

The third criterion was the factor structure of the Mandarin Chinese CIS. The CFA showed evidence for the unidimensional structure of the Mandarin Chinese CIS, which compatible with previous studies (32, 33). However, some items had lower factor loadings (range 0.42–0.91), which were similar to the previous study (0.40–0.82) (33), but lower compared to the initial study (0.72–0.86) (32). While some researchers have proposed a threshold value of 0.5 for factor loadings (46), the acceptable cutoff for factor loading depends on the research’s specific purpose. For example, Stevens suggested that a factor loading greater than 0.40 is sufficient (48). To improve the usefulness of CIS, more research is necessary to provide more evidence on its factor structure in the future.

In addition to the limitations mentioned above, this study also has several other potential shortcomings. Firstly, the participants were exclusively college students, and the majority of them were Mandarin Chinese. As a result, the generalizability of the Mandarin Chinese CIS to other age groups could be limited. Secondly, the study only evaluated the Mandarin Chinese CIS’s criterion validity using the DASS-21, FCV-19S, and SWLS as comparison tools. Future research should include a clinical sample to investigate whether the Mandarin Chinese CIS can effectively differentiate between individuals with and without COVID-19. The third limitation of the study was that participants scored relatively low on the Mandarin Chinese assessment. This may have been due to the timing of data collection. On January 8th, 2023, the Chinese government classified COVID-19 infections as “Class B management” (49). As a result, the majority of college students have already been infected with the COVID-19 and are in an immune period at the time of the study. Nevertheless, our study still identified the unique predictive power of Mandarin Chinese CIS for negative emotional states. Future research could corroborate our findings by examining the predictive power of Mandarin Chinese CIS in high periods of the COVID-19, such as during winter. The fourth limitation of the study was the imbalance in the distribution of male and female participants, particularly evident in the test–retest assessments. Our survey collection process was voluntary and anonymous, making it difficult to predict or control for potential gender differences in response rates. Despite our efforts to recruit a representative sample, we encountered challenges that resulted in the gender imbalance. To address this issue, it is necessary for future research to implement targeted recruitment strategies in order to ensure a more balanced representation of male and female participants.

## Data availability statement

The raw data supporting the conclusions of this article will be made available by the authors, without undue reservation.

## Ethics statement

The studies involving humans were approved by the Institutional Review Board at Shandong Youth University of Political Science. The studies were conducted in accordance with the local legislation and institutional requirements. The participants provided their electronic informed consent to participate in this study.

## Author contributions

QZ: Conceptualization, Formal analysis, Funding acquisition, Writing – original draft. YL: Investigation, Formal analysis, Writing – review & editing. JY: Conceptualization, Writing – review & editing. CL: Data curation, Formal analysis, Writing – original draft. HY: Investigation, Writing – review & editing.

## Footnotes

^1^ https://www.wjx.cn/
